# Lysosome-associated membrane glycoprotein 1 predicts fratricide amongst T cell receptor transgenic CD8^+^ T cells directed against tumor-associated antigens

**DOI:** 10.18632/oncotarget.10647

**Published:** 2016-07-18

**Authors:** Andreas Kirschner, Melanie Thiede, Franziska Blaeschke, Günther H.S. Richter, Julia S. Gerke, Michaela C. Baldauf, Thomas G.P. Grünewald, Dirk H. Busch, Stefan Burdach, Uwe Thiel

**Affiliations:** ^1^ Laboratory for Functional Genomics and Transplantation Biology, Departments of Pediatrics and Children's Cancer Research Center, Klinikum rechts der Isar, Technische Universität München, Munich, Germany; ^2^ Laboratory for Immunotherapy, Dr. von Hauner Children's Hospital, Medical center of the LMU Munich, Munich, Germany; ^3^ Laboratory for Pediatric Sarcoma Biology, Institute of Pathology of the LMU Munich, Munich, Germany; ^4^ German Cancer Consortium (DKTK), Heidelberg, Germany; ^5^ German Cancer Research Center (DKFZ), Heidelberg, Germany; ^6^ Institute for Medical Microbiology, Immunology and Hygiene, Technische Universität München, Munich, Germany

**Keywords:** fratricide, CD107a, TCR transgenic T cells, cross reactivity, amino-acid exchange scan

## Abstract

**Aim:**

Autologous as well as allogeneic CD8^+^ T cells transduced with tumor antigen specific T cell receptors (TCR) may cause significant tumor lysis upon adoptive transfer. Besides unpredictable life-threatening off-target effects, these TCRs may unexpectedly commit fratricide. We hypothesized lysosome-associated membrane glycoprotein 1 (LAMP1, CD107a) to be a marker for fratricide in TCR transgenic CD8^+^ T cells.

**Methods:**

We identified HLA-A*02:01/peptide-restricted T cells directed against ADRB3^295^. After TCR identification, we generated HLA-A*02:01/peptide restricted TCR transgenic T cells by retroviral transduction and tested T cell expansion rates as well as A*02:01/peptide recognition and ES killing in ELISpot and xCELLigence assays. Expansion arrest was analyzed via Annexin and CD107a staining. Results were compared to CHM1^319^-TCR transgenic T cells.

**Results:**

Beta-3-adrenergic receptor (ADRB3) as well as chondromodulin-1 (CHM1) are over-expressed in Ewing Sarcoma (ES) but not on T cells. TCR transgenic T cells demonstrated HLA-A*02:01/ADRB3^295^ mediated ES recognition and killing in ELISpot and xCELLigence assays. 24h after TCR transduction, CD107a expression correlated with low expansion rates due to apoptosis of ADRB3 specific T cells in contrast to CHM1 specific transgenic T cells. Amino-acid exchange scans clearly indicated the cross-reactive potential of HLA-A*02:01/ADRB3^295^- and HLA-A*02:01/CHM1^319^-TCR transgenic T cells. Comparison of peptide motive binding affinities revealed extended fratricide among ADRB3^295^ specific TCR transgenic T cells in contrast to CHM1^319^.

**Conclusion:**

Amino-acid exchange scans alone predict TCR cross-reactivity with little specificity and thus require additional assessment of potentially cross-reactive HLA-A*02:01 binding candidates. CD107a positivity is a marker for fratricide of CD8^+^ TCR transgenic T cells.

## INTRODUCTION

Adoptive T cell transfer has gained importance in the treatment of cancer. During the last 25 years, several approaches have been clinically introduced with variable results [1–6]. The infusion of unmodified autologous tumor infiltrating lymphocytes as well as the use of *in vivo* immune-stimulation using immune-checkpoint inhibitors [1–6] showed impressive responses e.g. in a number of melanoma and lung cancer patients. This effect may be limited to melanoma patients due to specific CD8^+^ T cell responses against immunogenic somatic mutations [7–10]. Attempts to translate autologous adoptive T cell transfer into the treatment of other solid cancer entities have yielded controversial results so far [3, 11–14].

Allogeneic stem cell transplantation is an established treatment for leukemia where donor T cells induce a graft-vs-leukemia response that can eradicate residual malignant cells [15], and is being explored as a treatment for a variety of other hematologic and non-hematologic malignancies [16, 17]. However, the infusion of unmodified donor lymphocyte infusion (DLI) after allogeneic stem cell transplantation may be associated with antitumor responses but is bought with a high risk of life threatening graft-versus-host disease (GvHD) [18].

In the search of specific and less toxic immune-therapeutic approaches, the introduction of genetically modified T cells transduced with a specific receptor (TCR) against tumor associated antigens essential for tumor survival has yielded promising (pre-) clinical results [5, 19–22]. However, cross-reactivity of these cells even against supposed cancer testis antigens could not be sufficiently predicted and remains a major concern in the clinical implementation [23–25]. Furthermore, the generation of viable TCR transgenic T cells may be hampered due to target expression in CD8^+^ T cells leading to fratricide [26].

Ewing sarcoma (ES) is a highly aggressive malignant pediatric bone tumor, which is still associated with poor outcome, especially in metastatic disease [27, 28]. It is characterized by pathognomonic chromosomal translocations fusing the *EWSR1* gene to various members of the *ETS* family of transcription factors, most commonly *FLI1* (85% of cases) [29]. EWSR1-FLI1 encodes an aberrant transcription factor that binds DNA at GGAA-microsatellites (mSats), which are converted by this protein to active enhancers [30]. EWSR1-FLI1-binding to GGAA-mSats drives the expression of oncogenic key downstream effectors [31, 32]. Previously, we identified different over-expressed genes in ES relative to normal tissues such as beta-3-adrenergic receptor (ADRB3) as well as chondromodulin-1 (CHM1), which may thus constitute attractive targets for HLA-A2/peptide allorestricted T cell therapy [33, 34]. In a previous work, we successfully generated HLA-A*02:01/CHM1^319^ transgenic TCR specific T cells, which killed ES cell lines *in vitro* and in a preclinical mouse model [35].

Lysosome-associated membrane protein 1 (LAMP1/CD107a) is a transmembrane protein and has shown to be a specific marker for degranulation for active T cells upon target recognition [36].

Here, we evaluate suitability of CD107a in combination with annexin positivity as a marker for fratricide of CD8^+^ TCR transgenic T cells. Furthermore, we assess the role of amino-acid exchange scans to predict cross-reactivity of HLA-A*02:01/ADRB3^295^- versus HLA-A*02:01/CHM1^319^-TCR transgenic T cells.

## RESULTS

### ADRB3 is over-expressed in ES

**Figure 1:**
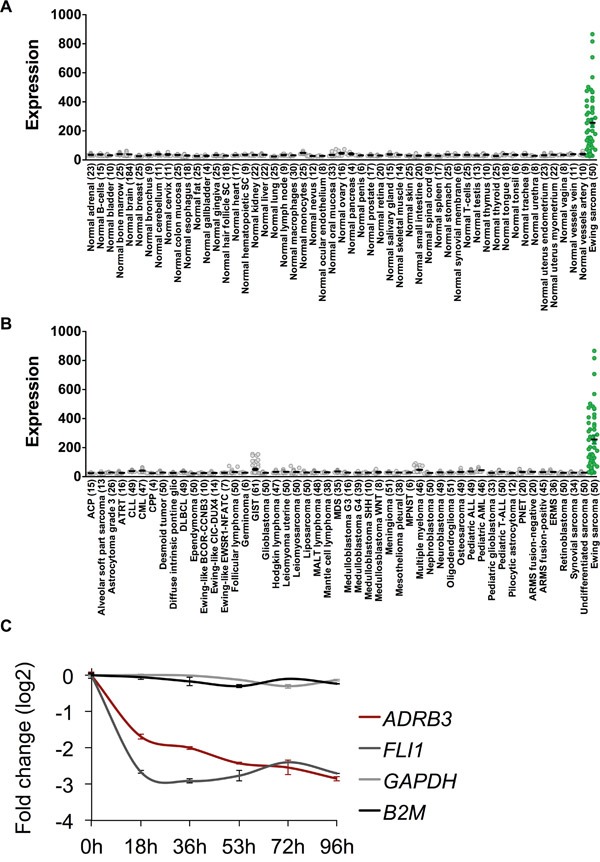
ADRB3 is an ES associated target antigen **A.** ADRB3 is over-expressed in Ewing Sarcoma compared to a Normal Body Atlas and **B.** compared to other tumor entities. **C.** Doxycyclin-induced knockdown of EWSR1-FLI1 in shows an EWSR1-FLI1 dependent expression of ADRB3 in A673/TR/shEF1 cells [37, 52].

We determined relative ADRB3 expression in ES samples compared to a normal body map, which included GvHD sensitive tissues such as colon mucosa and retina (Figure 1A). Further we compared ADRB3 expression with various tumor entities showing its exclusive expression in ES (Figure 1B). Chip-Seq analysis for SK-N-MC and A673 showed an EWSR1-FLI1 binding to a GGAA-microsatellite with activating enhancer-marks close to the ADRB3 gene (Supplementary Figure 1). RNAi-mediated downregulation of EWSR1-FLI1 confirmed a correlation of ADRB3 expression with EWSR1-FLI1 (Figure 1C).

### Peptide selection and identification of HLA-A*02:01/ADRB3^295^ TCR transgenic T cells

**Figure 2:**
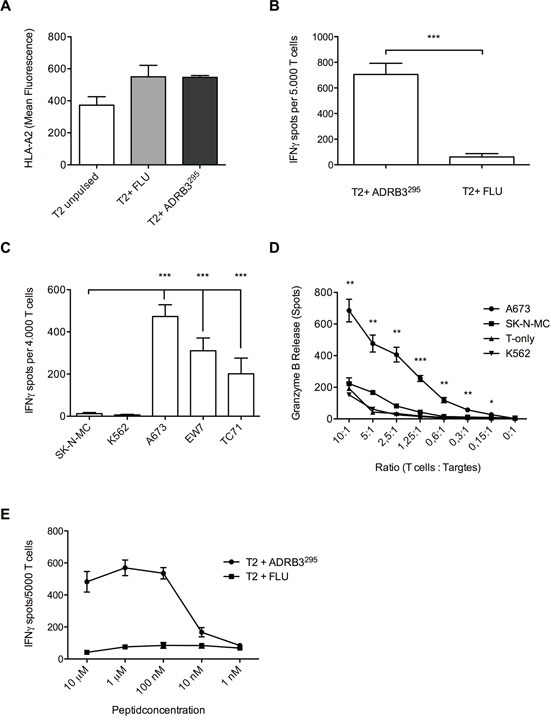
Wild type T cell clone ADRB3-1F4 specifically recognizes and kills HLA-A*02:01/ADRB3^295^ presenting ES cell lines **A.** Flow-cytometry confirms HLA-A*02:01/ADRB3^295^ binding on T2 cells. ADRB3-1F4 shows peptide specificity and HLA-A*02:01 restriction against **B.** peptide loaded T2 cells and against **C.** HLA-A*02:01/ADRB3^+^ ES cell lines compared to controls in IFNγ ELISpot assays. **D.** Dose-dependent killing of HLA-A*02:01/ADRB3^+^ ES cell lines compared to controls determined by ELISpot granzyme B release. **E.** T cell clone ADRB3-1F4 recognizes ADRB3^295^ peptide pulsed on T2 cells in an IFNγ ELISpot assay in a dose dependent manner. IFNγ release diminishes at a threshold of <0.01 μM. Data are presented as mean and SEM. A673, EW7 and TC-71; HLA-A*02:01^+^ ES, SK-N-MC: HLA-A*02:01^−^ ES, K562: MHC^−^ NK cell control. Error bars represent standard deviation of triplicate experiments. Asterisks indicate significance levels. p values < 0.05 were considered statistically significant (*p < 0.05; **p < 0.005; ***p < 0.0005).

Peptides with high *in silico* binding probabilities were selected and analyzed for HLA-A*02:01 stabilization on T2 cells via flow-cytometry (Figure 2A). The influenza matrix nonamer (FLU, GILGFVFTL) served as a positive control. ADRB3^295^ (GLIMGTFTL) was considered as a suitable HLA-A*02:01 binding peptide and was chosen for subsequent T cell priming.

The ADRB3-1F4 T cell clone recognized ADRB3^295^ loaded T2 cells in contrast to irrelevant influenza peptide loaded T2 cells (Figure 2B). ELISpot analyses revealed specific recognition of HLA-A*02:01^+^/ADRB3^+^ ES cell lines A673, TC-71, and EW7 versus the HLA-A*02:01^−^/ADRB3^+^ ES cell line SK-N-MC (Figure 2C). Granzyme B ELISpots revealed specific and dose-dependent killing of A673 versus SK-N-MC and K562 (Figure 2D). Further, T2 peptide titration showed dose-dependency of T cell activity and high binding affinity of the TCR towards the selected peptide in contrast to irrelevant influenza peptide loaded T2 cells (Figure 2E).

### Functional activity of HLA-A*02:01/ADRB3^295^ TCR transgenic T cells

**Figure 3:**
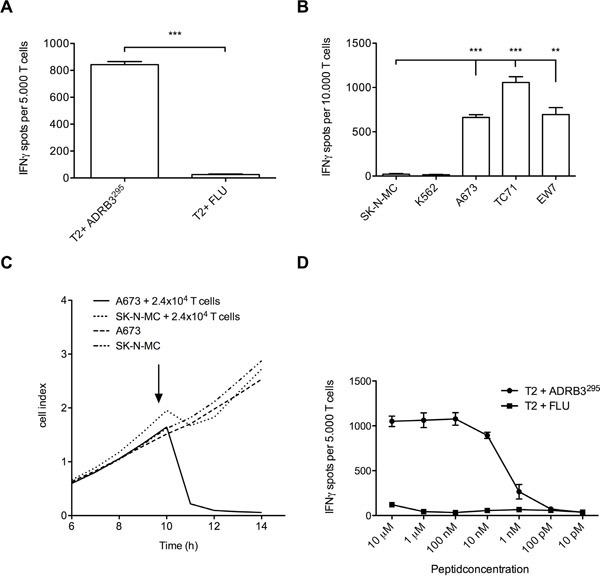
Functional evaluation of ADRB3^295^-TCR transgenic T cells **A.** ADRB3^295^-TCR transgenic T cells specifically recognize ADRB3^295^ pulsed T2 cells and **B.** HLA-A*02:01/ADRB3^+^ ES cell lines in contrast to controls. **C.** ADRB3^295^-TCR transgenic T cells specifically kill HLA-A*02:01/ADRB3^+^ ES cell line A673 in an xCELLigence assay compared to controls. **D.** ADRB3^295^-TCR transgenic T cells recognize ADRB3^295^ peptide pulsed on T2 cells in an IFNγ ELISpot assay in a dose dependent manner. IFNγ release diminishes at a threshold of <0.01 μM. Data are presented as mean and SD. A673, EW7 and TC-71; HLA-A*02:01^+^ ES, SK-N-MC: HLA-A*02:01^−^ ES, K562: MHC^−^ NK cell control. E/T ratio for ELISpot assay: (A and D) 1:4, (B) 1:2. Error bars represent standard deviation of triplicate experiments. Asterisks indicate significance levels. *p < 0.05; **p < 0.005; ***p < 0.0005.

We successfully identified the ADRB3-1F4 TCR sequence via PCR and FACS (Supplementary Figure 2A & 2B). HLA-A*02:01/ADRB3^295^ TCR transgenic T cells generated after retroviral transfer were isolated via magnetic beads and cultured after isolation (Supplementary Figure 3). IFN-γ ELISpot assays confirmed specific recognition of ADRB3^295^ loaded T2 cells and ES target cell lines by the TCR transgenic T cells (Figure 3A and 3B). Specific lysis of the ES cell line A673 was shown via real time analysis of target cell detachment in xCELLigence assays. After addition of HLA-A*02:01/ADRB3^295^ TCR transgenic T cells only A673 was lysed compared to SK-N-MC. Control cell line growth SK-N-MC was unaffected by the added TCR transgenic T cells (Figure 3C). An additional T2 peptide titration re-confirmed equal avidity of the transgenic TCR compared to the native TCR (Figure 3D).

### HLA-A*02:01/ADRB3^295^ TCR transgenic T cells are positive for CD107a when causing fratricide after transduction

**Figure 4:**
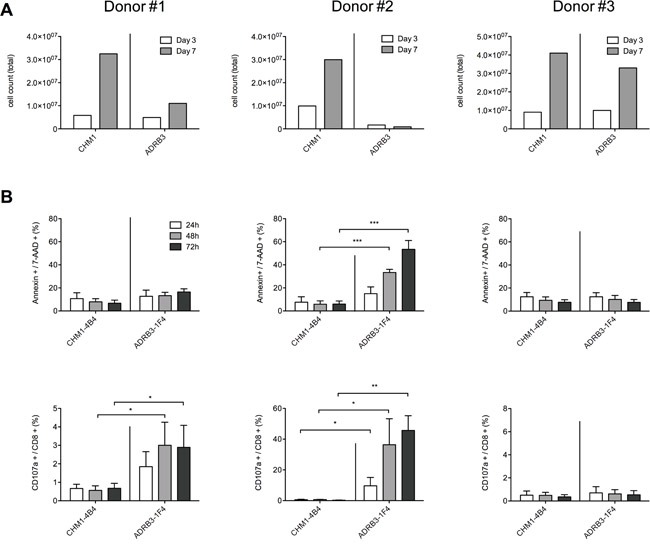
CD107a/annexin positivity is a sensitive early marker of ADRB3^295^-TCR transgenic T cell committed fratricide **A.** In PBMC donors #1 and #2 ADRB3^295^-TCR transgenic T cell expansion is hampered compared to CHM1^319^-TCR transgenic T cells as measured by total cell counts at expansion day 3 and day 7, respectively; **B.** Flow cytometry reveals correlation of CD107a/annexin expression with expansion impairment of ADRB3^295^-TCR transgenic T cells in contrast to CHM1^319^-TCR transgenic T cells. Asterisks indicate significance levels. *p < 0.05; **p < 0.005; ***p < 0.0005.

We assessed TCR transduction with three different donors (Donor#1 being HLA-A01; -A03, Donor#2 HLA-A02, -A11, and Donor#3 HLA-A26, -A29 positive) and measured T cell activity, apoptosis and T cell expansion over time. In two out of three different PBMC donors ADRB3^295^-TCR transgenic T cell expansion was hampered compared to CHM1^319^-TCR transgenic T cells as measured by total cell counts at expansion day 3 and day 7, respectively (Figure 4A). Flow cytometry revealed correlation of CD107a/annexin positivity with expansion impairment of ADRB3^295^-TCR transgenic T cells in contrast to CHM1^319^-TCR transgenic T cells (Figure 4B). For Donor#1 only CD107a but no annexin positivity was measurable 72 h after transduction. However, annexin increased after 7 days, which correlated with the impaired cell growth. CD107a was as well still detectable for Donor#1 (Supplementary Figure 4A + 4B). We further assessed to scan for further suitable donors using CD107a as a marker to predict fratricide. Incubation of ADRB3^295^- and CHM1^319^-TCR transgenic T cells with various donor PBMCs only revealed one new possible donor where no CD107a was measurable. For CHM1 no limitations were cognizable (Supplementary Figure 4C)

### Amino-acid exchange scans insufficiently predict cross-reactivity of CD8^+^ TCR transgenic T cells and require additional HLA-A*02:01 binding prediction

**Figure 5:**
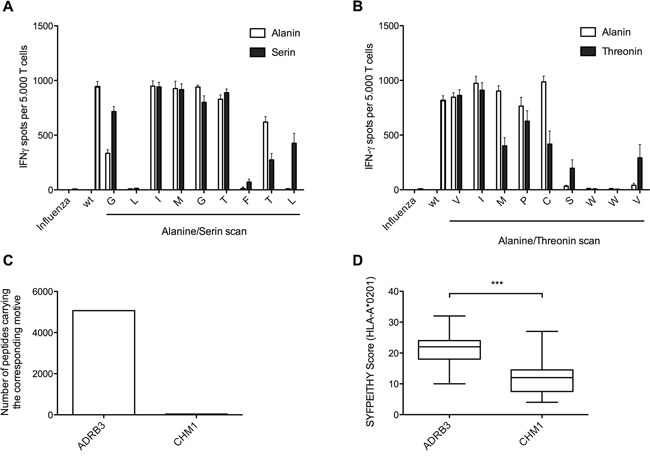
Amino-acid exchange scans yield only limited power to predict cross-reactivity of CD8^+^ TCR transgenic T cells in contrast to *in silico* HLA-A*02:01 binding prediction **A + B.** ADRB3^295^-TCR transgenic T cells as well as CHM1^319^-TCR transgenic T cells reveal unspecific recognition upon selective amino-acid exchange of respective target antigens to (A) alanine/serine and (B) alanine/threonine in IFNγ ELISpot assays. **C.** The number of peptides carrying the ADRB3 specific core motive is much higher in comparison to peptides carrying the CHM1 specific core motive. **D.** Peptides with similar ADRB3^295^ motive patterns (n=780) show higher binding scores for HLA-A*02:01 in comparison to the peptides with similar CHM1^316^ motive (n=33). *p < 0.05; **p < 0.005; ***p < 0.0005.

As expression analysis for ADRB3 in PBMCs proved negative, we excluded antigen expression as the cause of T cell mediated fratricide (Supplementary Figure 5A). ADRB3^295^-TCR (Figure 5A) as well as CHM1^319^-TCR (Figure 5B) transgenic T cells demonstrate unspecific recognition upon selective amino-acid exchange of respective target antigens to alanine/serine and alanine/threonine in IFNγ ELISpot assays. *In silico* analyses identified 5068 different peptides that carried the ADRB3^295^ motive X-L-X-X-X-X-F-X-[LA] in contrast to 38 different peptides carrying the X-X-X-X-X-[ST]-W-W-[VT] CHM1^319^ motive (Figure 5C). The high amount of ADRB3^295^ related peptides already indicates an increased cross reactivity. Additionally, *in silico* predicted binding strength to HLA-A*02:01 (Figure 5D), HLA-A*01, and HLA-A*03 (Supplementary Figure 5B + 5C) revealed higher binding scores for ADRB3 derived peptides in comparison to CHM1. This high accessibility of high binding peptides for different HLAs further confirms our observation of fratricide upon transduction in an allogeneic setting.

### HLA-A*02 blocking of ES target cell lines further gave insight in ADRB3^295^ TCR cross reactivity

**Figure 6:**
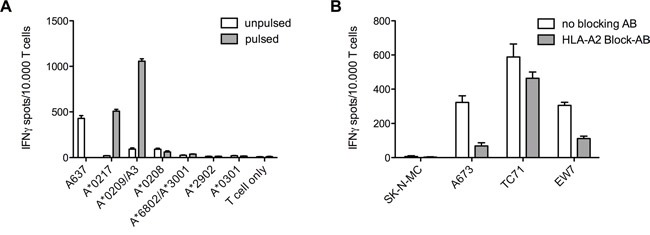
Various LCL cell lines and HLA-A*02 blocking of ES target cell lines to distinguish T cell cross-reactivity **A.** Recognition of LCLs was limited to two lines expressing the HLA-A*02 superfamily. Further pulsing the cells with specific ADRB3^295^ peptide also gave no further hints for cross-reactivity. ES cell line A673 served as a positive control. **B.** Blocking of the HLA-A*02 molecules on ES cell lines shows reduced IFNγ release in ELISpot assays. E/T ratio for ELISpot assay: 1:2. Error bars represent standard deviation of triplicate experiments.

Further assessment for cross-reactivity of the ADRB3^295^ specific TCR with various LCL cell lines only HLA-A*02 positive cell lines were recognized. Additional pulsing of these cells with ADRB3^295^ peptide only increased T cell recognition of two cell lines (Figure 6A). An additional HLA-A*02 blockage of the ES target cell lines however was able to show extended reactivity of the TCR (Figure 6B).

## DISCUSSION

Adoptive immunotherapy with autologous or allogeneic donor T cells against tumor-associated antigens has proven a promising tool to eradicate residual disease resistant to conventional therapy regimens [5, 15, 20].

The transfusion of cancer antigen specific CARs (chimeric antigen receptors) or TCR transduced T cells demonstrated curative potential in the treatment of cancer [4]. However, the potential of putative tumor specific immune cells to cross-react and to cause severe and fatal off-target effects remains a major concern in the clinical implementation [23–25, 48]. Several approaches to predict cross-reactivity mediated on-/off target activity have been proposed [49–51] but even the use of supposedly cancer/testis specific antigens could not exclude the possibility of fatal toxicity following adoptive transfer [48]. Furthermore, even weak tumor associated antigen expression on TCR transgenic T cells is sufficient to induce cytotoxic responses within the T cell population leading to an *in vitro* expansion failure - a phenomenon that has been termed fratricide [26]. In a previous work, we successfully generated HLA-A*02:01/CHM1^319^ transgenic TCR specific T cells, which killed ES cell lines *in vitro* and in a preclinical mouse model [35].

In this work we aimed at generating TCR transgenic T cells specific against an antigen derived from ADRB3 that we previously identified as being specifically up-regulated in ES [33]. Identification, isolation and expansion of ADRB3 specific T cells from healthy donors were successful. However, after TCR identification, cloning and retroviral transduction into donor T cells we observed hampered expansion rates. Most attempts to generate viable T cells failed 7 days after transduction in contrast to the expansion rates of previously established CHM1^319^-TCR transgenic T cells. Several studies identified low target antigen expression on TCR transduced T cells as the cause for artificially induced fratricide [26]. After verification that ADRB3 is not expressed on CD8^+^ T cells, we hypothesized that fratricide could occur due to cross-reactivity based on peptide resemblance of ADRB3^295^ and yet unidentified non-tumor associated antigens presented on the T cell surface. CD107a as well as annexin expression is known to be associated with apoptosis [26, 36]. We demonstrate that CD107a/annexin expression is an early marker to indicate fratricide within a T cell population transduced with a TCR against a putative tumor associated target antigen ADRB3^295^. In contrast, CD107a/annexin negative CHM1^319^-TCR transgenic T cells were easily expandable. Alanine/serine as well as alanine/threonine exchange scans, however, clearly predicted cross-reactivity of TCR against both ADRB3^295^ and CHM1^319^ albeit cross-reactivity was slightly weaker for CHM1^319^. An additional analysis of the respective recognition patterns was necessary to illuminate the reasons for the disparate TCR reactivity between both specificities. The number and the binding affinity of *in silico* predicted motive patterns similar to the respective original peptide sequence, e.g. ADRB3^295^ *versus* CHM1^319^ correlated with the probability of the TCR to commit fratricide. When we further assessed cross-reactivity for ADRB3^295^ specific T cells using different LCLs or by blocking HLA-A*02, the LCL cell lines here were not suited to confirm the mayor cross-reactivity we were observing. However, by blocking HLA in IFNγ ELISpot assays we could show T cell cross-reactivity, which is restricted to certain HLAs as we already could see in our transduction results.

T cell mediated immunotherapy is a promising tool to fight cancer. However, as it remains a challenge to clearly predict the cross-reactive and thus life-threatening potential of candidate TCRs directed against putative tumor specific antigens *in vitro*, it is necessary to develop tools to determine a risk-threshold of permissible TCR promiscuity prior to its introduction into the clinical setting. Identification of supposedly suitable tumor specific TCRs is a laborious and time-consuming procedure with unpredictable clinical potential [48]. In this work, amino-acid exchange scans of candidate target antigens seem not sufficient to determine TCR suitability. They harbor the risk to erroneously exclude curative tumor specific TCRs due to over interpretation of background cross reactivity to a small number of antigens of minor clinical importance. We wish to emphasize the importance to correlate the results of such amino-exchange scans with the number of potential motive patterns of the respective target peptide sequences and their respective *in silico* binding scores. In order to facilitate the clinical implementation of engineered cancer specific T cells and to minimize the risk of T cell mediated toxicity in the future, a consentient scoring system based on a broad spectrum of *in vitro* and *in silico* predictive tools is warranted, including CD107a/annexin staining.

## MATERIALS AND METHODS

### Cell lines

SK-N-MC and TC-71 (both ES cell lines) were obtained from the German Collection of Microorganisms and Cell Cultures (DSMZ; Braunschweig, Germany). A673 (ES cells) were obtained from ATCC (LGC Standards GmbH, Wesel, Germany). The EW7 ES cell line was obtained from Olivier Delattre, Institut Curie, Paris. The TAP-deficient HLA*A02:01^+^ T2 cell line (somatic cell hybrid) was obtained from P. Cresswell (Yale University School of Medicine, New Haven, CT, USA). The HLA-A*02:01^−^ erythroid leukemia cell line K562 was a gift from A. Knuth and E. Jäger (Krankenhaus Nordwest, Frankfurt, Germany). All cell lines were routinely tested for purity and mycoplasma contamination. Lymphoblastoid cell lines (LCL) were provided by A. Krackhardt (Klinikum rechts der Isar, TU München). HLA genotyping was done at the Labor für Immungenetik und Molekulare Diagnostik (LMU, Munich). Tumor cell lines were cultured in RPMI 1640 supplemented with 10 % fetal calf serum (FCS, Biochrom, Berlin, Germany), 100 U/ml penicillin, 100 μg/ml streptomycin, and 2 mM L-glutamine (all from Life Technologies). RPMI 1640 medium for LCL and T2 cells was supplemented with 1 mM sodium pyruvate and non-essential amino acids, additionally.

### Analysis of microarray data

Publicly available gene expression data were downloaded from the Gene Expression Omnibus or the European Bioinformatics Institute and were either generated on the Affymetrix HG-U133A2 microarray (EWSR1-FLI1 knockdown time-course experiment; GSE27524) [37] or on the Affymetrix HG-U133Plus2.0 microarray (primary tumors and normal-body-map) [38]. Microarray data were rigorously quality-checked and normalized – separately for each chip type – simultaneously using RMA and brainarray CDF files (v19, ENTREZG) yielding one optimized probe-set per gene [39]. The comparative analysis of primary tumors and normal tissues comprised 50 tumor entities and 71 normal tissue types. Data accession codes: GSE10927, GSE31048, E-MTAB-1940, GSE11504, GSE7307, GSE26457, GSE14461, GSE27678, GSE8671, GSE3526, GSE41168, GSE43346, GSE16134, GSE44765, GSE18676, GSE19429, GSE11151, GSE40231, GSE40791, GSE2125, GSE7158, GSE53223, GSE20986, GSE30784, GSE18520, GSE12621, GSE13355, GSE14926, GSE25518, GSE33630, GSE68015, GSE13433, GSE4290, GSE19404, GSE39671, GSE13159, GSE58697, GSE26576, GSE53786, GSE21687, GSE34620, GSE34800, GSE60740, GSE53820, GSE19348, GSE17743, GSE53733, GSE17920, GSE13319, GSE21050, GSE13314, GSE36000, GSE29326, GSE37418, GSE4780, E-MTAB-1719, GSE19784, GSE53224, GSE16476, E-MEXP-3628, GSE47051, GSE17855, GSE19578, GSE39816, GSE50161, E-TABM-1202, GSE29683, GSE20196, GSE19750, GSE30522, GSE63626, GSE22780, GSE28133, GSE40611, GSE25550, GSE6338, GSE32569, GSE35493, GSE8167, GSE30195, GSE14827, GSE46170, GSE61352, GSE63941.

### Analysis of DNase-seq and ChIP-seq data

Publicly available DNase-seq and ChIP-seq data was performed as previously described [32]. Briefly, data were retrieved from the GEO. ENCODE [40] SK-N-MC DNase-seq (GSM736570) were analysed within the Nebula environment [41] using Model-based Analysis of ChIP-Seq v1.4.2 (MACS) [42] and converted to *. wig format for display in the UCSC Genome Browser [43]. Pre-processed ChIP-seq data from Riggi et al. (GSE61944) [30] were converted from the *. bigwig to the *. wig format using the UCSC bigWigToWig conversion tool. Accession codes: GSM1517569, GSM1517572, GSM1517544, GSM1517553, GSM1517548, GSM1517557, GSM1517547, GSM1517556, GSM1517570, GSM1517573.

### Isolation of PBMCs

Peripheral blood mononuclear cells (PBMCs) were isolated from human peripheral blood samples of healthy donors (obtained with IRB approval and informed consent from the DRK-Blutspendedienst Baden-Württemberg-Hessen in Ulm, Germany) by centrifugation over Ficoll-Paque (GE Healthcare, Freiburg, Germany) according to the supplier's recommendations.

### Generation of dendritic cells (DCs)

CD14^+^ cells were isolated from PBMCs with anti-human CD14 magnetic particles (BD Biosciences, Heidelberg, Germany) according to the manufacturer's instructions. Purity of cells was confirmed by flow cytometry on a FACS Calibur (BD Bioscience). Culture and maturation of CD14^+^ cells was done as described previously [34].

### Isolation of CD8^+^ T cells

CD8^+^ T cells were isolated from human HLA-A*02:01^−^ PBMCs by negative isolation using a cocktail of biotin-conjugated non-CD8 monoclonal antibodies and anti-biotin micro beads followed by column depletion according to manufacturer's instructions (Miltenyi Biotec, Bergisch Gladbach, Germany). Purity of isolated CD8^+^ T cells was confirmed by flow cytometry.

### *In vitro* priming of HLA-A*02:01/ADRB3^295^ allo-restricted T cells

Mature DCs were re-suspended in T-cell medium (AIM-V supplemented with 5 % human AB serum, 2 mM L-glutamine, and 50 μL ml-1 gentamycin) and pulsed with selected peptides at a concentration of 30 – 50 μM in the presence of 20 μg ml-1 β1MG (Sigma, Taufkirchen, Germany) for 4 h at 37°C and 5 % CO_2_. Pulsed cells were than washed and used for T cell priming as described previously [34].

### Multimer-staining and cell sorting

Two weeks after *in vitro* priming activated T cells were pooled and stained with specific peptide/HLA-A*02:01-multimer-PE and CD8-FITC (BD Bioscience) for cell sorting. An unspecific peptide/HLA-A*02:01-multimer-PE directed against LIPI (Lipase member I, LLNEEDMNV) served as a negative control (Dirk Busch, Technical University Munich) [44]. Cell sorting was done on a FACS Aria (BD Bioscience).

### Limiting dilution

After FACS sorting, multimer-PE specific T cells were expanded using limiting dilution. Expansion was conducted in round-bottom 96-well plates in 200 μl T cell medium supplemented with anti-CD3 (30 ng ml-1), rhIL-2 (100 U ml-1), rhIL-15 (2 ng ml-1); irradiated LCL (1 × 10^5^ per well) and irradiated PBMCs pooled from three different donors (5 × 10^4^ per well) were used as feeder as previously described [34]. Cytokines and 100 μl medium / well were replaced after 1 week. Expanded T cells were further characterized in ELISpot assays.

### Vβ analysis of T-cell receptor repertoire

To determine T cell clonality and Vβ expression, the IOTest Beta Mark Kit (Beckman Coulter, Brea, CA, USA) was used according to the manufacturer's protocol. This kit is designed for flow cytometric determination of the T cell repertoire (TCR) and covers about 70 % of the normal human TCR Vβ repertoire.

### ELISpot assay

96-well mixed cellulose ester plates (MultiScreen-HA Filter Plate, 0,45 μm Millipore, Eschborn, Germany) and capture-antibody solutions (all Mabtech, Hamburg, Germany) were used for IFNγ and granzyme B ELISpot assay as described previously [34]. Spots in plates were counted on an AID-ELIRIFL04 ELISpot reader (Autoimmun Diagnostika, Strassberg, Germany). All experiments were performed in triplets with exception of the initial screening ELISpot.

### xCELLigence proliferation assay

Cell proliferation was measured with an impedance-based instrument system (xCELLigence, Roche/ACEA Biosciences) enabling label-free real time analysis. Briefly, 1 × 10^4^ to 2.5 × 10^4^ targets cells were seeded in 200 μl medium. During the exponential growth phase 100 μl was replaced by a 100 μl T cell suspension. Cellular impedance was measured periodically every 15 min after T cell addition.

### Identification of TCR sequence

Primers for the identification of the TCR were used according to Schuster et al. [45]. RNA from T cell clones was isolated via TRI Reagent Solution (Invitrogen). For cDNA synthesis the High Capacity cDNA Reverse Transcription Kit (Applied Biosystems) was used according to manufactures protocol. TCR PCR was carried out using the AccuPrime™ Taq DNA Polymerase System (Invitrogen) and an Eppendorf Master Cycler. PCR reaction was done in twin. tec real-time PCR plate 96 (Eppendorf). Primers, PCR composition and cycler setting are listed in Supplementary Tables 1 - 4. PCR samples were loaded onto 1.5 % agarose gels and run at 110 V for 50 min. 1 KB Plus DNA Ladder (Life Technologies) was used for size determination. PCR products at the expected sizes (370 – 500 bp for alpha chain and 190 – 290 bp for beta chain) were isolated with the StrataPrep Gel Extraction Kit (Agilent) and sent for sequencing (Sequiserve, Vaterstetten). Sequencing identified parts of the alpha and beta chains. New primers were implemented according to the predicted TCR sequence by IMGT/V-QUEST covering the whole sequence of the according alpha and beta chain (Supplementary Table 5). Sequence modifications were done to improve expression via codon optimization and minimal murinization for the ADRB3-1F4 TCR [46]. Both chains were linked via a P2A sequence (Supplementary Table 6). This construct was than synthesized and cloned into the MP71 vector (done by Gene Art, Life Technologies, Regensburg).

### Transduction and isolation of CD8^+^ T cells

293T GalV virus producing cells were seeded at a concentration of 0.2 × 10^6^ / well in 3 ml DMEM onto 6 well plates 24 hours prior to transfection. Transfection was performed using TransIT-293T according to manufacturer's manual. 200 μl of serum-free medium was placed into a 1.5 ml FACS tube. 9 μl of TransIT were added, vortexed, and incubated at RT for 20 min. 1 μg of TCR plasmid was added and mixed carefully. After 30 min incubation, the solution was added drop-wise onto the cells and incubated for 48 h at 37°C. Virus containing supernatant was collected, centrifuged at 1,000 g for 5 min and sterile filtered (0.45 μm). Virus was used fresh or stored at −80°C. PBMCs / T cells for viral transduction were isolated from Buffy coats and stimulated with 50 ng / ml OKT-3 and 100 U / ml rhIL-2 48 h prior to spin infection. The day before transduction non-treated 24-well plates were coated with 400 μl Retronectin^®^ in PBS at a concentration of 12.5 μg / ml and stored at 4°C. Directly before transduction the supernatant was removed. Wells were blocked with 2 % BSA in PBS for 30 min at 37°C and washed twice with 2.5 % HEPES in HBSS. Stimulated PBMCs / T cells were collected and set to a concentration of 1 × 10^6^ / ml in TCM. 1 ml of each Virus and T cells were added into coated 24-well plates plus additional Protamine-sulfate (c_end_ = 4 μg / ml), HEPES (c_end_ = 0,5 %), and IL-2 (c_end_ = 100 U / ml). Plates were centrifuged for 90 min at 820 g in 32°C preheated centrifuge and stored at 37°C, 5 % CO_2_ over night. The next day cells were harvested and split 1 : 1. Cells were again placed on coated 24-well plates with fresh virus plus additives and centrifuged at 820 g / 90 min / 32°C. Medium was replaced after 48 h and transduction efficiency was checked after 72 h via FACS multimer staining. TCR transgenic T cells were isolated via magnetic anti-PE microbeads according to manufacturers manual (Miltenyi). Isolated cells were then cultured using irradiated mixed PBMCs and LCLs as feeder cells.

### CD107a staining

To show T cell activity via CD107a FACS staining after transduction T cells were taken directly out of the well and washed twice with ice-cold staining buffer and stained for CD8-APC, specific multimer-PE and CD107a-FITC in 100 μl FACS buffer. After 30 min incubation at 4°C, plates were washed twice and analyzed by flow-cytometry. To check for possible cross reactivity TCR transgenic T cells were incubated with Donor PBMCs for 2 h at 37°C in a ration of T cells to PBMCs 1 : 2 before staining in 96-well plates.

### Annexin staining

To assess T cell apoptosis after transduction the PE Annexin V Apoptosis Detection Kit I (BD) was used according to the manufacturers manual. Briefly, cells were washed twice with PBS and re-suspended in binding buffer at a concentration of 1 × 10^6^ / ml. 100 μl of cell suspension were stained with 5 μl of PE Annexin V and 5 μl 7-AAD and mixed. After 15 min incubation at RT 400 μl of 1x binding buffer was added and cells were analyzed by flow-cytometry within 1 h.

### Amino-acid exchange scans

In order to verify HLA-A*02:01/peptide TCR specificity alanine/serine or alanine/threonine substituted peptides were introduced subsequently replacing each position of the original amino acid sequence (Supplementary Table 7). T2 cells were pulsed for 2 hours with these peptides prior to IFN-γ ELISpot assays. The amount of ADRB3^295^ and CHM1^319^ peptide (each 0.01 μM) used for this assay was chosen according to the corresponding peptide titration curves. Substituted amino acid positions causing non-recognition were considered crucial for TCR/pMHC interaction. In contrast, amino acid exchanges without effect on T cell recognition were marked by an ‘X’. Peptide motives were analyzed using the ExPASy ScanProsite tool. Peptides with similar recognition patterns represent possible off-target antigens and were further controlled for *in silico* predicted MHC binding strength by use of SYFPEITHI [47].

### Statistical analysis

Descriptive statistics were used to determine mean and standard deviation of the mean (SD). Differences were analyzed by unpaired two-tailed student's t-test using either Excel (Microsoft) or Prism 5 (GraphPad Software); p values < 0.05 were considered statistically significant (*p < 0.05; **p < 0.005; ***p < 0.0005).

## SUPPLEMENTARY MATERIALS FIGURES AND TABLES




