# Supplementary material for: Lysosome-associated membrane glycoprotein 1 predicts fratricide amongst T cell receptor transgenic CD8+ T cells directed against tumor-associated antigens
Source: Oncotarget. 2016 Jul 18;7(35):56584–97. doi: 10.18632/oncotarget.10647 (PMC5302936; doi:10.18632/oncotarget.10647)
Supplement: Supplementary file 1 [file oncotarget-07-56584-s001.pdf]

Lysosome-associated membrane glycoprotein 1 predicts fratricide amongst T cell receptor transgenic CD8+ T cells directed against tumor-associated antigens

SUPPLEMENTARY FIGURES AND TABLES

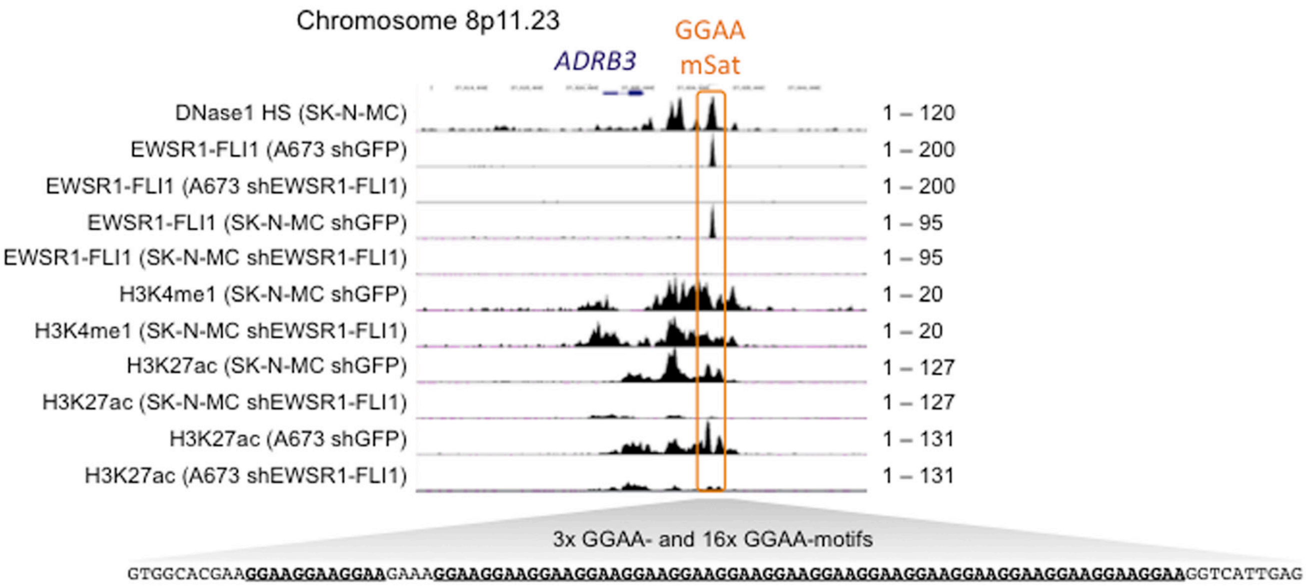

Supplementary Figure S1: Analysis of published DNase-Seq and ChIP-Seq data revels a prominent peak to a GGAA-microsatellit close to the *ADRB3* gene. Displayed signal from published DNase-Seq and ChIP-Seq data in Ewing Sarcoma cells transfected with either a control shRNA (shGFP) or a specific shRNA against EWSR-FLI1 [30]. Readout is given on the right. The depicted genomic sequence correspond to the human reference sequence (hg19).

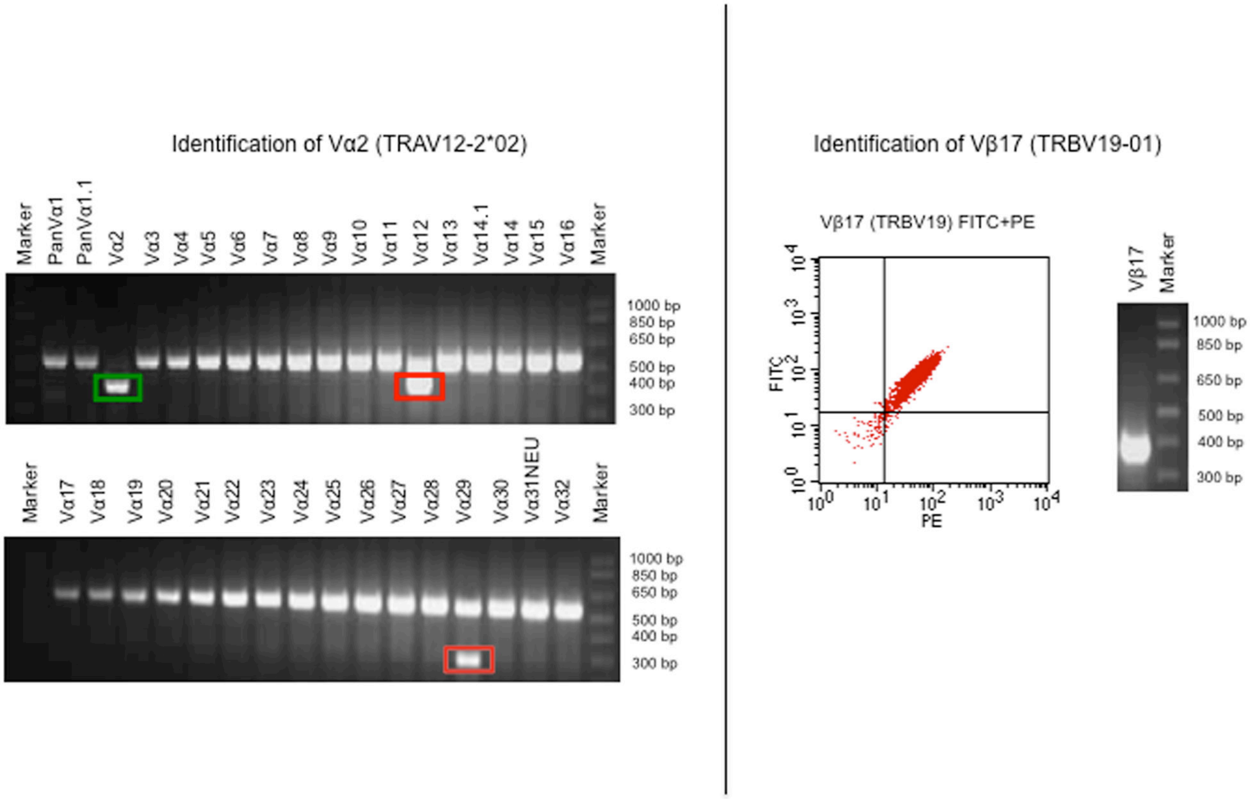

Supplementary Figure S2: Wildtype ADRB3-1F4 TCR is clonal. A. ADRB3-1F4 was only positive for the variable α-chain TRAV12-2*02 and the variable β-chain TRBV19-01 (detected by the Vα2 primer and by the Vβ17 primer, respectively) and was considered clonal. All other sequenced PCR products were unspecific or showed no open reading-frame (red bordered boxes). **B.** Clonality and Vβ chain expression of the T cell clone was shown via the IOTest Beta Mark Kit.

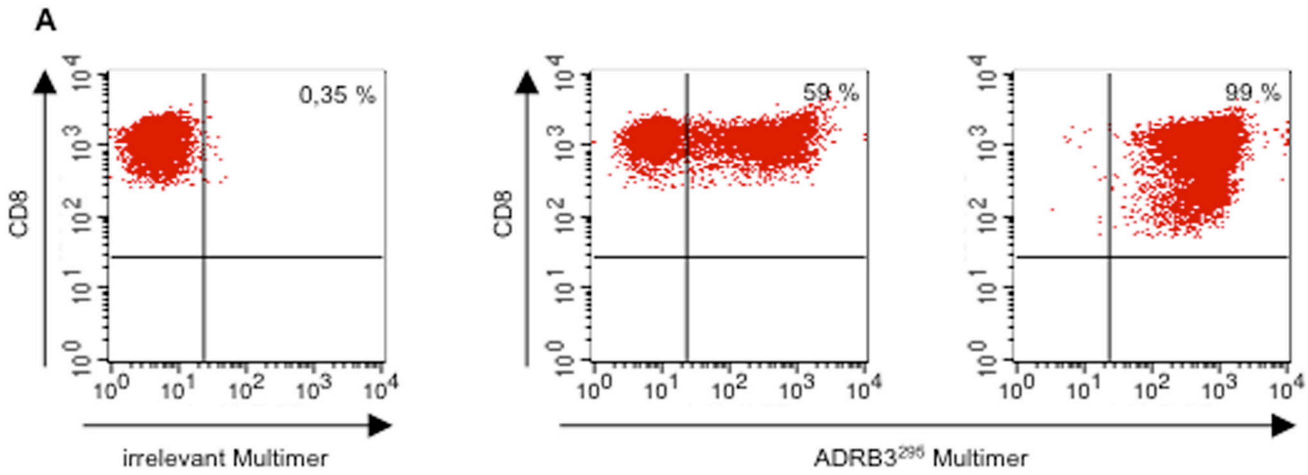

Supplementary Figure S3: Transduction and isolation efficiency. A. Flow cytometry reveals a transduction rate of 59% for ADRB3[295] TCR transgenic T cells. Purification using HLA-A*02:01-ADRB3[295]-multimer staining reveals a 99% ADRB3[295]-TCR transgenic T cell purification after magenitc bead separation. An irrelevant multimer serves as control (live gate).

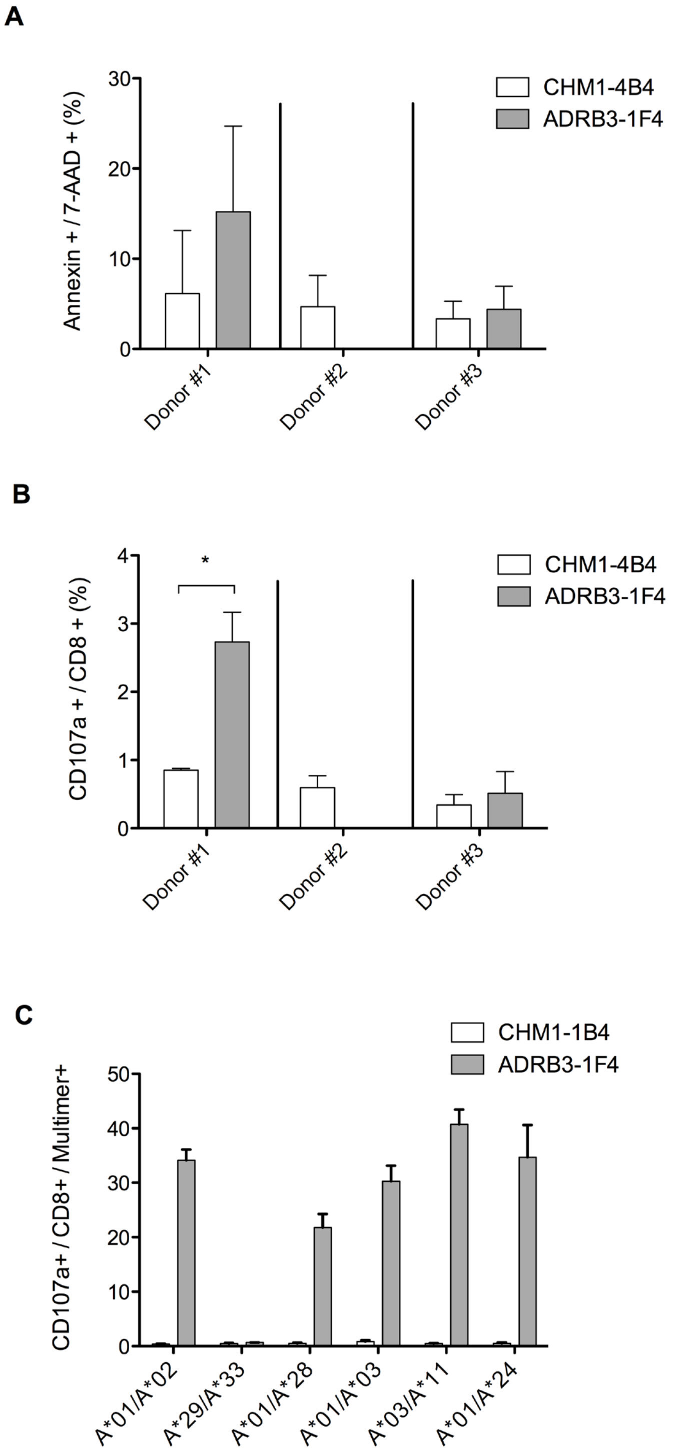

Supplementary Figure S4: Determination of apoptosis and CD107a 7 days after transduction. A + B. At day 7 after TCR transduction apoptosis is increased in ADRB3^{295}-TCR transgenic T cells of donor #1 in comparison to CHM1^{319}-TCR transgenic T cells as indicated by annexin and CD107a expression. ADRB3^{295}-TCR transgenic T cells in donor #2 had vanished 7 days after transduction. **C.** Incubation of TCR transgenic T cells shows only one suitable donor for transduction with ADRB3^{295} whereas CHM1^{319} shows no limitations. Error bars represent standard deviation of triplicate experiments.

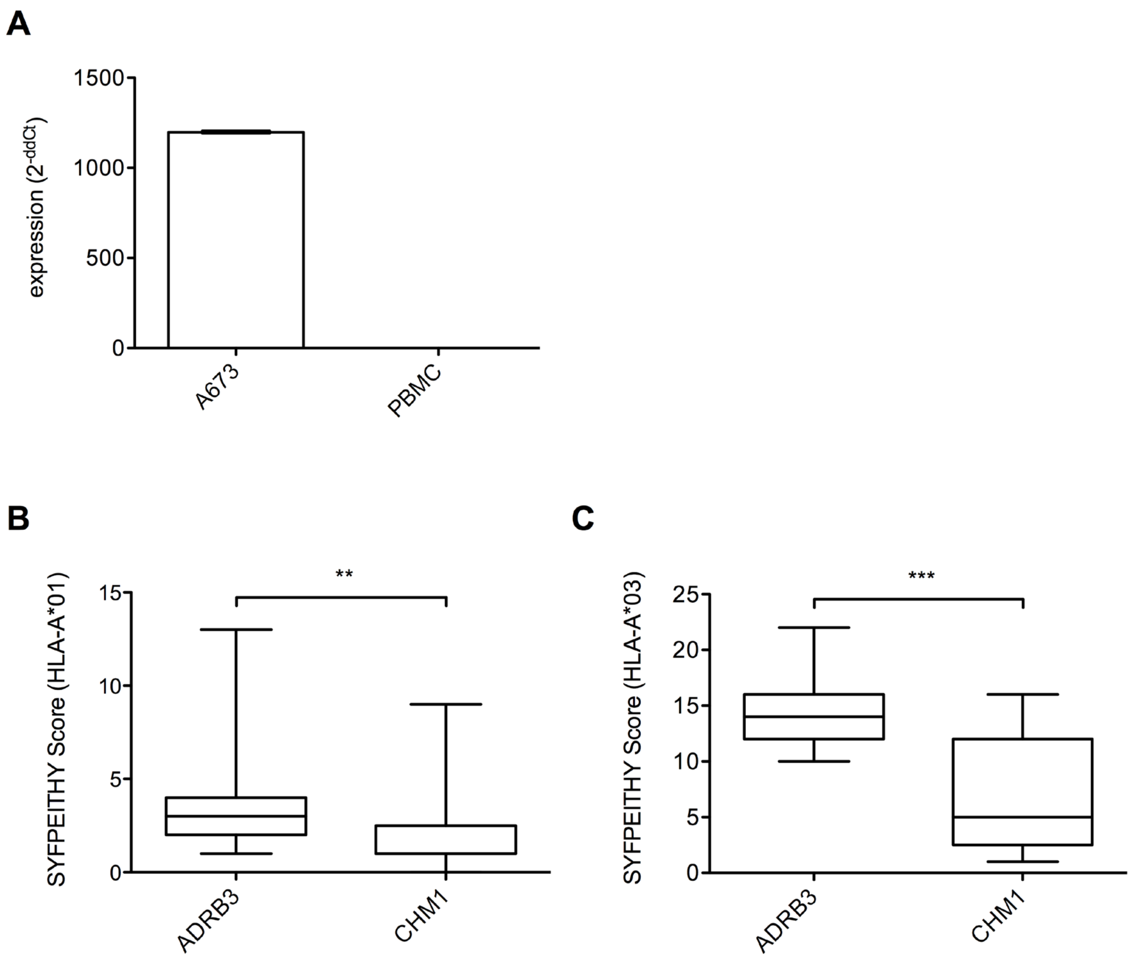

Supplementary Figure S5: Comparison of *in silico* predicted binding strength for HLA-A*01 and HLA-A*03. A. ADRB3 expression was not detected in isolated PBMCs **B + C.** Peptides with similar ADRB3[295] motive patterns (n=180) show higher binding scores for HLA-A*01 (left) and HLA-A*03 (right) in comparison to peptides with similar CHM1[316] motive (n=33). *$p < 0.05$; **$p < 0.005$; ***$p < 0.0005$.

Supplementary Table S1: Primers for the identification of Vα chain

See Supplementary File 1

Supplementary Table S2: Primers for the identification oft the Vβ chain

See Supplementary File 1

Supplementary Table S3: Specific primers for the identification oft he ADRB3-1F4 TCR

**Specific primers for the ADRB3-1F4 TCR:**	
TRAV12-2*02	ATG ATG AAA TCC TTG AGA GTT TTA C
TRBV19*03	CAA CCA GGT GCT CTG CTG T
**PCR Master Mix for the detection of the TCR β-chain:**	
DEPC-H_2O	18,5 µl
Puffer (10x)	2,5 µl
P-5’βST (2,5 pmol/µl)	0,5 µl
P-3’bST (2,5 pmol/µl)	0,5 µl
3’CβII (5 pmol/µl)	1,0 µl
cDNA	0,5 µl
Polymerase	0,5 µl
**PCR Master Mix for the detection of the TCR α-chain:**	
DEPC-H_2O	18,5 µl
Puffer (10x)	2,5 µl
P-5’αST (2,5 pmol/µl)	0,5 µl
P-3’αST (2,5 pmol/µl)	0,5 µl
3’T-Cα (5 pmol/µl)	1,0 µl
cDNA	0,5 µl
Polymerase	0,5 µl

Supplementary Table S4: Setting of the PCR cycler

The thermocycler was programmed as followed:			
**Step**		**Temp**	**Duration**
1	Initial denaturation	94°C	6 min
2	Denaturation	94°C	1 min
3	Annealing	54°C	1 min
4	Elongation	68°C	1 min
5	Final elongation	68°C	7 min
6	Cooling	4°C	∞

Steps 2 to 4 were repeated 40 times.

ADRB3-1F4 TCR sequences. ADRB3-1F4wt is the wildtype β- and α-chain linked via a P2A element. ADRB3-1F4mm is the corresponding TCR sequence after minimal murinization and codon optimization.

Supplementary Table S5: Sequence of the wild type ADRB3-1F4 TCR sequence linked via a P2A element

TCR Sequences	ADRB3-1F4 wild type
ADRB3-1F4wt	ATGAGCAACCAGGTGCTCTGCTGTGTGGTCCTTTGTTTCCTGGGAGCAAACA<br>CCGTGGATGGTGGAATCACTCAGTCCCCAAAGTACCTGTTCAGAAAGGAAGG<br>ACAGAATGTGACCCTGAGTTGTGAACAGAATTTGAACCACGATGCCATGTACT<br>GGTACCGACAGGACCCAGGGCAAGGGCTGAGATTGATCTACTACTCACAGATA<br>GTAAATGACTTTCAGAAAGGAGATATAGCTGAAGGGTACAGCGTCTCTCGGGAG<br>AAGAAGGAATCCTTTCCTCTCACTGTGACATCGGCCCAAAAGAACCCGACAGCTT<br>TCTATCTCTGTGCCAGTACTACAACGGGGGTGAATGAGCAGTTCTTCGGGCCAGGG<br>ACACGGCTCACCGTGCTAGAAGATCTGCGGAACGTGACCCCCCCTAAGGTGTCCCTGT<br>TCGAGCCCAGCAAGGCCGAGATCGCCAACAAGCAGAAAGCCACCCTGGTCTGCCT<br>GGCTAGGGGCTTCTTCCCCGACCACGTGGAGCTGTCTTGGTGGGTGAACGGCAAAGAG<br>GTGCACAGCGGCGTCAGCACCGACCCACAGGCCTACAAAGAGAGCAACTACAG<br>CTACTGCCTGTCCTCTAGACTGCGGGTGTGCGCCACCTTCTGGCACAACCCCCGGA<br>ACCACTTCCGGTGCCAGGTGCAGTTCCACGGCCTGAGCGAAGAGGACAAGTGGC<br>CCGAGGGCAGCCCCAAGCCCGTGACACAGAACATCAGCGCCGAGGCCTGGGGCAGAG<br>CCGACTGCGGCATCACCAGCGCCAGCTACCACCAGGGCGTGCTGTCTGCCACCATC<br>CTGTACGAGATCCTGCTGGGCAAGGCCACCCTGTACGCCGTGCTGGTGTCCGGCCTG<br>GTGCTGATGGCCATGGTGAAGAAGAAGAACAGCGGCAGCGGCGCCACCAACTTCAGC<br>CTGCTGAAACAGGCCGGCGACGTGGAAGAGAACCCTGGCCCTATGATGAAATCC<br>TTGAGAGTTTTACTAGTGATCCTGTGGCTTCAGTTGAGCTGGGTTTGGAGCC<br>AACAGAAGGAGGTGGAGCAGAATTCTGGACCCCTCAGTGTTCCAGAGGGAGCC<br>ATTGCCTCTCTCAACTGCACTTACAGTGACCGAGGTTCCCAGTCCTTCTTCTGGTA<br>CAGACAATATTCTGGGAAAAGCCCTGAGTTGATAATGTCCATATACTCCAATG<br>GTGACAAAGAAGATGGAAGGTTTACAGCACAGCTCAATAAAGCCAGCCAGTATGTTT<br>CTCTGCTCATCAGAGACTCCCAGCCCAGTGATTCAGCCACCTACCTCTGTGCCGTGG<br>GTAACGACTACAAGCTCAGCTTTGGAGCCGGAACCACAGTAACTGTAAGAGCAAA<br>TATCCAGAACCCCGAGCCCGCCGTGTACCAGCTGAAGGACCCCAGATCTCAGGACT<br>CTACACTGTGCCTGTTCACCGACTTCGACAGCCAGATCAACGTGCCCAAGACC<br>ATGGAAAGCGGCACCTTCATCACCGACAAGACCGTGCTGGACATGAAGGCCATG<br>GACAGCAAGAGCAACGGCGCCATTGCCTGGTCCAATCAGACCAGCTTCACATG<br>CCAGGACATCTTCAAAGAGACAAACGCCTGCTACCCCAGCTCCGACGTGCCCTGCGAC<br>GCCACCCTGACCGAGAAGAGCTTCGAGACAGACATGAACCTGAATTTCCAGAACCTGAG<br>CGTGATGGGCCTGAGGATCCTGCTGCTGAAGGTGGCCGGCTTCAATCTGCTGATGACCCTG<br>CGGCTGTGGAGCAGCTGA

Supplementary Table S6: Sequence oft he pMP71-ADRB3-1F4mm TCR sequence with modifications and codon optimization.

TCR Sequences ADRB3-1F4 after minimal murinization and codon optimization

ADRB3-1F4mm	ATGAGCAACCAGGTGCTCTGCTGTGTGGTCCTTTGTTTCCTGGGAGCAAACA<br>CCGTGGATGGTGGAATCACTCAGTCCCCAAAGTACCTGTTCAGAAAGGAAGG<br>ACAGAATGTGACCCTGAGTTGTGAACAGAATTTGAACCACGATGCCATGTACT<br>GGTACCGACAGGACCCAGGGCAAGGGCTGAGATTGATCTACTACTCACAGATA<br>GTAAATGACTTTCAGAAAGGAGATATAGCTGAAGGGTACAGCGTCTCTCGGGAG<br>AAGAAGGAATCCTTTCCTCTCACTGTGACATCGGCCCAAAAGAACCCGACAGCTTT<br>CTATCTCTGTGCCAGTACTACAACGGGGGTGAATGAGCAGTTCTTCGGGCCAGGGACAC<br>GGCTCACCGTGCTAGAGGACCTGAAAAACGTGTTCCCACCCGAGGTCGCTGTGTT<br>TGAGCCATCAAAGGCAGAGATCGCTCACACCCAAAAGGCCACACTGGTGTGCCTGGCC<br>ACAGGCTTCTACCCCGACCACGTGGAGCTGAGCTGGTGGGTGAATGGGAAGGAGGTGC<br>ACAGTGGGGTCAGCACAGACCCGCAGCCCCTCAAGGAGCAGCCCGCCCTCAATGACTCC<br>AGATACTGCCTGAGCAGCCGCCTGAGGGTCTCGGCCACCTTCTGGCAGAACCCCCGCAA<br>CCACTTCCGCTGTCAAGTCCAGTTCTACGGGCTCTCGGAGAATGACGAGTGGACCCA<br>GGATAGGGCCAAACCTGTCACCCAGATCGTCAGCGCCGAGGCCTGGGGTAGAGCAGAC<br>TGTGGCATAACCTCCGCTTCTTACCATCAAGGGGTCCTGTCTGCCACCATCCTCTATGAG<br>ATCTTGCTAGGGAAGGCCACCTTGTATGCCGTGCTGGTCAGTGCCCTCGTGCTGATGG<br>CCATGGTCAAGAGAAAGGATTCCAGAGGCGGCAGCGGCGCCACCAACTTCAGCC<br>TGCTGAAACAGGCCGGCGACGTGGAAGAGAACCCTGGCCCTATGATGAAATCCTTGAG<br>AGTTTTACTAGTGATCCTGTGGCTTCAGTTGAGCTGGGTTTGGAGCCAACAGAAGGAGGT<br>GGAGCAGAATTCTGGACCCCTCAGTGTTCCAGAGGGAGCCATTGCCTCTCTCAACTGCAC<br>TTACAGTGACCGAGGTTCCCAGTCCTTCTTCTGGTACAGACAATATTCTGGGAAAAGCCC<br>TGAGTTGATAATGTCCATATACTCCAATGGTGACAAAGAAGATGGAAGGTTTACA<br>GCACAGCTCAATAAAGCCAGCCAGTATGTTTCTCTGCTCATCAGAGACTCCCAGCCCAG<br>TGATTCAGCCACCTACCTCTGTGCCGTGGGTAACGACTACAAGCTCAGCTTTGGAGCC<br>GGAACCACAGTAACTGTAAGAGCAAATATCCAGAACCCTGACCCTGCCGTGTACCAGC<br>TGAGAGACTCTAAATCCAGTGACAAGTCTGTCTGCCTATTCACCGATTTTGATTCTCAA<br>ACAAATGTGTCACAAAGTAAGGATTCTGATGTGTATATCACAGACAAAACTGTGCTAG<br>ACATGAGGTCTATGGACTTCAAGAGCAACAGTGCTGTGGCCTGGAGCA<br>ACAAATCTGACTTTGCATGTGCAAACGCCTTCAACAACAGCATTATTCCAGAAG<br>ACACCTTCTTCCCCAGTGACGTCCCTTCCTGTGATGTCAAGCTGGTCGAG<br>AAAAGCTTTGAAACAGATACGAACCTAAACTTTCAAAACCTGTCAGTGATTGGGTT<br>CCGAATCCTCCTCCTGAAAGTGGCCGGGTTTAATCTGCTCATGACGCTGCGGCTGTGGT<br>CCAGCTGA

List of used peptides for the alanine/serin or alanine/threonine scan. Position of peptide modification is indicated. Peptides with substitutions were ordered with a purity of at least 70 %. ADRB3295 and CHM1319 wild type peptides had a purity of > 90 %.

Supplementary Table S7: Used wt peptides and AA exchange peptides for loading T2 cells

See Supplementary File 1
