# Supplementary material for: Lysosome-associated membrane glycoprotein 1 predicts fratricide amongst T cell receptor transgenic CD8+ T cells directed against tumor-associated antigens
Source: Oncotarget. 2016 Jul 18;7(35):56584–97. doi: 10.18632/oncotarget.10647 (PMC5302936; doi:10.18632/oncotarget.10647)
Supplement: Supplementary file 2 [file oncotarget-07-56584-s002.docx]

**T cell α-chain repertoire primer** (Steinle, Reinhardt et al. 1995)

| P-5’αST | ST CTG TGC TAG ACA TGA GGT CT |  |
| --- | --- | --- |
| P-3’αST | ST CTT GCC TCT GCC GTG AAT GT |  |
| 3’T-Cα | GGT GAA TAG GCA GAC AGA CTT GTC ACT GGA |  |
| PANVα1 | AGA GCC CAG TCT GTG ASC CAG | S = C/G |
| PANVα1.1 | AGA GCC CAG TCR GTG ACC CAG | R = A/G |
| Vα2 | GTT TGG AGC CAA CRG AAG GAG |  |
| Vα3 | GGT GAA CAG TCA ACA GGG AGA |  |
| Vα4 | TGA TGC TAA GAC CAC MCA GC |  |
| Vα5 | GGC CCT GAA CAT TCA GGA |  |
| Vα6 | GGT CAC AGC TTC ACT GTG GCT A |  |
| Vα7 | ATG TTT CCA TGA AGA TGG GAG |  |
| Vα8 | TGT GGC TGC AGG TGG ACT |  |
| Vα9 | ATC TCA GTG CTT GTG ATA ATA |  |
| Vα10 | ACC CAG CTG CTG GAG CAG AGC CCT |  |
| Vα11 | AGA AAG CAA GGA CCA AGT GTT |  |
| Vα12 | CAG AAG GTA ACT CAA GCG CAG ACT |  |
| Vα13 | GAG CCA ATT CCA CGC TGC G |  |
| Vα14.1 | CAG TCC CAG CCA GAG ATG TC |  |
| Vα14 | CAG TCT CAA CCA GAG ATG TC |  |
| Vα15 | GAT GTG GAG CAG AGT CTT TTC |  |
| Vα16 | TCA GCG GAA GAT CAG GTC AAC |  |
| Vα17 | GCT TAT GAG AAC ACT GCG T |  |
| Vα18 | GCA GCT TCC CTT CCA GCA AT |  |
| Vα19 | AGA ACC TGA CTG CCC AGG AA |  |
| Vα20 | CAT CTC CAT GGA CTC ATA TGA |  |
| Vα21 | GTG ACT ATA CTA ACA GCA TGT |  |
| Vα22 | TAC ACA GCC ACA GGA TAC CCT TCC |  |
| Vα23 | TGA CAC AGA TTC CTG CAG CTC |  |
| Vα24 | GAA CTG CAC TCT TCA ATG C |  |
| Vα25 | ATC AGA GTC CTC AAT CTA TGT TTA |  |
| Vα26 | AGA GGG AAA GAA TCT CAC CAT AA |  |
| Vα27 | ACC CTC TGT TCC TGA GCA TG |  |
| Vα28 | CAA AGC CCT CTA TCT CTG GTT |  |
| Vα29 | AGG GGA AGA TGC TGT CAC CA |  |
| Vα30 | GAG GGA GAG AGT AGC AGT |  |
| Vα31NEU | TCG GAG GGA GCA TCT GTG ACT A |  |
| Vα32 | CAA ATT CCT CAG TAC CAG CA |  |

Table 1 Primers for the identification of Vα chain

**T cell β-chain repertoire primer** (Steinle, Reinhardt et al. 1995)

| P-5βST | AAG CAG AGA TCT CCC ACA C |  |
| --- | --- | --- |
| P-3βST | GAG GTA AAG CCA CAG TCT GCT |  |
| P-3CβII | GAT GGC TCA AAC ACA GCG ACC TC |  |
| Vβ1 | GCA CAA CAG TTC CCT GAC TTG GCA C |  |
| Vβ2 | TCA TCA ACC ATG CAA GCC TGA CCT |  |
| Vβ3 | GTC TCT ACA TAT GAG AGT GGA TTT GTC ATT |  |
| Vβ5.1 | ATA CTT CAG TGA GAC ACA GAG AAA C |  |
| Vβ5.2 | TTC CCT AAC TAT AGC TCT GAG CTG |  |
| Vβ6.1 | GCC CAG AGT TTC TGA CTT ACT TC |  |
| Vβ6.2 | ACT CTG ASG ATC CAG CGC ACA | S=C/G |
| Vβ6.3 | ACT CTG AAG ATC CAG CGC ACA |  |
| Vβ7 | CCT GAA TGC CCC AAC AGC TCT C |  |
| Vβ8 | ATT TAC TTT AAC AAC AAC GTT CCG |  |
| Vβ8S3 | GCT TAC TTC CGC AAC CGG GCT CCT |  |
| Vβ9 | CCT AAA TCT CCA GAC AAA GCT |  |
| Vβ10 | CTC CAA AAA CTC ATC CTG TAC CTT |  |
| Vβ11 | TCA ACA GTC TCC AGA ATA AGG ACG |  |
| Vβ12 | AAA GGA GAA GTC TCA GAT |  |
| Vβ12S3 | GCA GCT GCT GAT ATT ACA GAT |  |
| Vβ13 | TCG ACA AGA CCC AGG CAT GG |  |
| Vβ13.1 | CAA GGA GAA GTC CCC AAT |  |
| Vβ13.2 | GGT GAG GGT ACA ACT GCC |  |
| Vβ13S5 | ATA CTG CAG GTA CCA CTG GCA |  |
| Vβ14 | GTC TCT CGA AAA GAG AAG AGG AAT |  |
| Vβ15 | AGT GTC TCT CGA CAG GCA CAG GCT |  |
| Vβ16 | AAA GAG TCT AAA CAG GAT GAG TCC |  |
| Vβ17 | CAG ATA GTA AAT GAC TTT CAG |  |
| Vβ18 | GAT GAG TCA GGA ATG CCA AAG GAA |  |
| Vβ19 | CAA TGC CCC AAG AAC GCA CCC TGC |  |
| Vβ20 | AGC TCT GAG GTG CCC CAG AAT CTC |  |
| Vβß21 | AAA GGA GTA GAC TCC ACT CTC |  |
| Vβ22.1 | CAT CTC TAA TCA CTT ATA CT |  |
| Vβ22.2 | AAG TGA TCT TGC GCT GTG TCC CCA |  |
| Vβ22.3 | CTC AGA GAA GTC TGA AAT ATT CG |  |
| Vβ23 | GCA GGG TCC AGG TCA GGA CCC CCA |  |
| Vβ24 | ATC CAG GAG GCC GAA CAC TTC T |  |
| Vβ25 | TGA AAA TGT CTT TGA TGA AAC AG |  |
| Vβ26 | CCT AAC GGA ACG TCT TCC AC |  |
| Vβ27 | ATA CTG GAA TTA CCC AGA CAC |  |
| Vβ28 | TAC ACA ATT CCC AAG ACA CAG |  |

Table 2 Primers for the identification oft the Vβ chain

**Peptides used for amino-acid exchange**

| Description | AA Sequence |
| --- | --- |
| ADRB3^295^ | GLIMGTFTL |
| ADRB3^295^-A1 | **A**LIMGTFTL |
| ADRB3^295^-A2 | G**A**IMGTFTL |
| ADRB3^295^-A3 | GL**A**MGTFTL |
| ADRB3^295^-A4 | GLI**A**GTFTL |
| ADRB3^295^-A5 | GLIM**A**TFTL |
| ADRB3^295^-A6 | GLIMG**A**FTL |
| ADRB3^295^-A7 | GLIMGT**A**TL |
| ADRB3^295^-A8 | GLIMGTF**A**L |
| ADRB3^295^-A9 | GLIMGTFT**A** |
| ADRB3^295^-S1 | **S**LIMGTFTL |
| ADRB3^295^-S2 | G**S**IMGTFTL |
| ADRB3^295^-S3 | GL**S**MGTFTL |
| ADRB3^295^-S4 | GLI**S**GTFTL |
| ADRB3^295^-S5 | GLIM**S**TFTL |
| ADRB3^295^-S6 | GLIMG**S**FTL |
| ADRB3^295^-S7 | GLIMGT**S**TL |
| ADRB3^295^-S8 | GLIMGTF**S**L |
| ADRB3^295^-S9 | GLIMGTFT**S** |
|  |  |
| CHM1^319^ | VIMPCSWWV |
| CHM1^319^-A1 | **A**IMPCSWWV |
| CHM1^319^-A2 | V**A**MPCSWWV |
| CHM1^319^-A3 | VI**A**PCSWWV |
| CHM1^319^-A4 | VIM**A**CSWWV |
| CHM1^319^-A5 | VIMP**A**SWWV |
| CHM1^319^-A6 | VIMPC**A**WWV |
| CHM1^319^-A7 | VIMPCS**A**WV |
| CHM1^319^-A8 | VIMPCSW**A**V |
| CHM1^319^-A9 | VIMPCSWW**A** |
| CHM1^319^-T1 | **T**IMPCSWWV |
| CHM1^319^-T2 | V**T**MPCSWWV |
| CHM1^319^-T3 | VI**T**PCSWWV |
| CHM1^319^-T4 | VIM**T**CSWWV |
| CHM1^319^-T5 | VIMP**T**SWWV |
| CHM1^319^-T6 | VIMPC**T**WWV |
| CHM1^319^-T7 | VIMPCS**T**WV |
| CHM1^319^-T8 | VIMPCSW**T**V |
| CHM1^319^-T9 | VIMPCSWW**T** |

Table 7 Used wt peptides and AA exchange peptides for loading T2 cells
